# A new immunohistochemistry prognostic score (IPS) for recurrence and survival in resected pancreatic neuroendocrine tumors (PanNET)

**DOI:** 10.18632/oncotarget.7436

**Published:** 2016-02-17

**Authors:** Antonio Viúdez, Filipe L.F. Carvalho, Zahra Maleki, Marianna Zahurak, Daniel Laheru, Alejandro Stark, Nilofer Z. Azad, Christopher L. Wolfgang, Stephen Baylin, James G. Herman, Ana De Jesus-Acosta

**Affiliations:** ^1^ Department of Medical Oncology, The Sidney Kimmel Comprehensive Cancer Center at Johns Hopkins Medical Institutions, Baltimore, Maryland, USA; ^2^ Department of Medical Oncology, Complejo Hospitalario de Navarra-Instituto de Investigaciones Sanitarias de Navarra-IDISNA, Pamplona, Navarra, Spain; ^3^ Department of Pathology, The Johns Hopkins Medical Institutions, Baltimore, Maryland, USA; ^4^ The Division of Biostatistics and Bioinformatics, Johns Hopkins University School of Medicine, Baltimore, Maryland, USA

**Keywords:** pancreatic neuroendocrine tumor, MGMT, NDRG-1, PHLDA-3, immunohistochemistry, Pathology Section

## Abstract

Pancreatic neuroendocrine tumor (PanNET) is a neoplastic entity in which few prognostic factors are well-known. Here, we aimed to evaluate the prognostic significance of N-myc downstream-regulated gen-1 (NDRG-1), O6-methylguanine DNA methyltransferase (MGMT) and Pleckstrin homology-like domain family A member 3 (PHLDA-3) by immunohistochemistry (IHC) and methylation analysis in 92 patients with resected PanNET and follow-up longer than 24 months. In multivariate analyses, ki-67 and our immunohistochemistry prognostic score (IPS-based on MGMT, NDRG-1 and PHLDA-3 IHC expression) were independent prognostic factors for disease-free-survival (DFS), while age and IPS were independent prognostic factors for overall survival (OS). Our IPS could be a useful prognostic biomarker for recurrence and survival in patients following resection for PanNET.

## INTRODUCTION

Pancreatic neuroendocrine tumor (PanNET) represents up to 5% of all pancreatic tumors [1–4]. Although most patients have an indolent clinical course, some patients have a more aggressive disease resistant to most available treatments [5]. Surgical resection of the primary tumor and the metastases remains the only curative treatment. When complete surgical excision is achieved [6] local or distant organ recurrence occurs in approximately 20% and 35% of the cases, respectively [7]. To date, there is no role for adjuvant treatments and patients are followed with clinical and imaging surveillance [8].

The World Health Organization (WHO) and the European Neuroendocrine Tumor Society (ENETS) have established prognostic-oriented criteria based on pathological specimens [9,10]. Higher tumor grade and high proliferative index Ki-67 have been associated with a more aggressive clinical course. These pathological criteria help to determine therapeutic decisions in patients with unresectable or metastatic disease [11]. However, there is an unmet need for prognostic biomarkers that can stratify patients at high risk for recurrence after surgical resection and/or to select the best treatment from available options.

Whole exome sequencing of PanNET specimens revealed that around 15% of patients have mutations in the PI3K/Akt pathway (TSC2, PTEN and PIK3CA) [12]. Treatments with mTOR inhibitors and temozolomide-based combinations have demonstrated clinical efficacy in phase II and III clinical trials [13,14], but their use is limited to patients with symptomatic, unresectable, or metastatic disease [15].

MGMT (O6-methylguanine-DNA methyltransferase) is the DNA repair protein responsible for removing alkylation adducts from *O*6-position of guanine in DNA. Several studies have shown that MGMT promoter hypermethylation with subsequent loss of MGMT expression might play a role in modulating chemosensitivity to alkylating agents and is utilized as a predictive biomarker to response to these drugs, in particular to temozolomide [16]. In the setting of PanNET tumors, only one study found a striking correlation between the absence of MGMT expression by immunohistochemistry (IHC) and better clinical outcomes in a small group of patients treated with temozolomide-based regimens [17]. However, results fail to show a definitive role for MGMT as a prognostic biomarker in PanNET [18,19].

NDRG-1 (N-myc downstream-regulated gen-1) controls the negative feedback-loop between the phosphatase and tensin homologue (PTEN) and the phosphoinositide 3-kinase (PI3K) pathway [20], a balance that usually is lost in cancer and frequently disturbed in PanNET [12,21]. NDRG-1 protein expression can be silenced by abnormal DNA methylation of the gene promoter [22] and modify tumor aggressiveness. In low grade gliomas high NDRG-1 cytoplasmic expression is associated with better patients’ outcomes. It is still unknown if NDRG-1 expression has the same effect on clinical outcomes of patients with PanNET [23].

Similarly, PHLDA-3 (Pleckstrin homology-like domain family A member 3) is a tumor suppressor gene recently discovered in PanNET [24], and described to be associated to worse clinical outcomes by decreased expression secondary to loss of heterozygosity (LOH) and methylation. PHLDA-3 IHC expression has been evaluated in several solid malignant tumors [25–27], but its role in PanNET remains unknown.

Accordingly, our objectives are to retrospectively analyze the prevalence of MGMT, PHLDA-3 and NDRG-1 promoter methylation status along with their IHC expression in a large cohort of patients with resected PanNET, and to determine the role of these biomarkers in clinical outcomes after surgical resection.

## RESULTS

Between January 1998 and December 2010, a total of 365 patients underwent resection of primary PanNET at Johns Hopkins Hospital (Baltimore, United States of America). 166 patients met the inclusion criterion of the study which were a follow-up longer than 24 months or had tumor progression within the first 24 months following surgery. Among the patients included in the study, the first ninety-two patients (55.4%) were selected for gene and protein expression analyses. Baseline characteristics of patients included in our study are summarized in Table 1 and Supplementary Table S1.

**Table 1:** Demographic characteristics

Variable	N N=92 % (n)
**Age**	_50_ 56_64_ (57±12)
**Gender** **Female** **Male**	51 (47) 49 (45)
**Grade** **I** **II** **III**	80 (68) 14 (12) 6 (5)
**Type** **Insulinoma** **Glucagonoma** **VIPoma** **Gastrinoma** **Somatostatinoma** **Non-functional**	4 (4) 2 (2) 2 (2) 3 (3) 2 (2) 86 (79)
**Ki-67** **≤2** **3-19** **≥20**	58 (49) 37 (31) 5 (4)
**AJCC** **1A** **1B** **2A** **2B** **3** **4**	22 (20) 15 (14) 8 (7) 36 (33) 1 (1) 18 (16)
**Size (cm)**	_2_2_4_(4±3)
**Nodes Affected** **Yes** **No**	50(45) 50 (45)
**Margin** **R0** **R1** **R2**	85 (78) 13 (12) 2 (2)
**Vascular Invasion** **Yes** **No**	26 (22) 74 (64)
**Perineural Invasion** **Yes** **No**	39 (34) 61 (54)
**Period Recluiment** **1998-2001** **2001-2005** **2005-2009** **2009-2012**	17 (16) 23 (21) 25 (23) 35 (32)

Descriptive analyses for immunohistochemistry patterns are summarized in Table 2. No significant associations were observed between NDRG-1 pattern and MGMT IHC, ki-67 or PHLDA-3 score.

**Table 2:** Descriptive analysis based on methylation and IHC status

	N	% of 92 patients (n)
**MGMT** **Tumor** **Normal**	81 70	58 (53) 40 (37)
**NDRG-1** **Tumor** **Normal**	77 63	45 (41) 37 (34)
**PHLDA-3** **Tumor** **Normal**	81 68	60 (55) 37 (34)
**NDRG-1 IHC** **0** **1 (weak)** **2 (moderate)** **3 (strong)** **Global Positive**	88	11 (10) 12 (11) 26 (23) 50 (44) 88 (78)
**PHLDA-3 IHC** **0** **1 (≤ 5%)** **2 (6-50%)** **3 (≥ 51%)** **Global Positive**	82	18 (15) 23 (19) 20 (16) 39 (32) 82 (67)
**NDRG-1 pattern** **Diffuse** **Patched**	78	72 (56) 28 (22)
**MGMT IHC** **0** **1 (≤ 5%)** **2 (6-50%)** **3 (≥ 51%)** **Global Positive**	85	27 (23) 22 (19) 21 (18) 29 (25) 73 (62)

### Effect of clinical-pathological variables, NDRG-1, MGMT and PHLDA-3 expression on DFS (Disease Free-Survival) and OS (Overall Survival)

**Figure 1:**
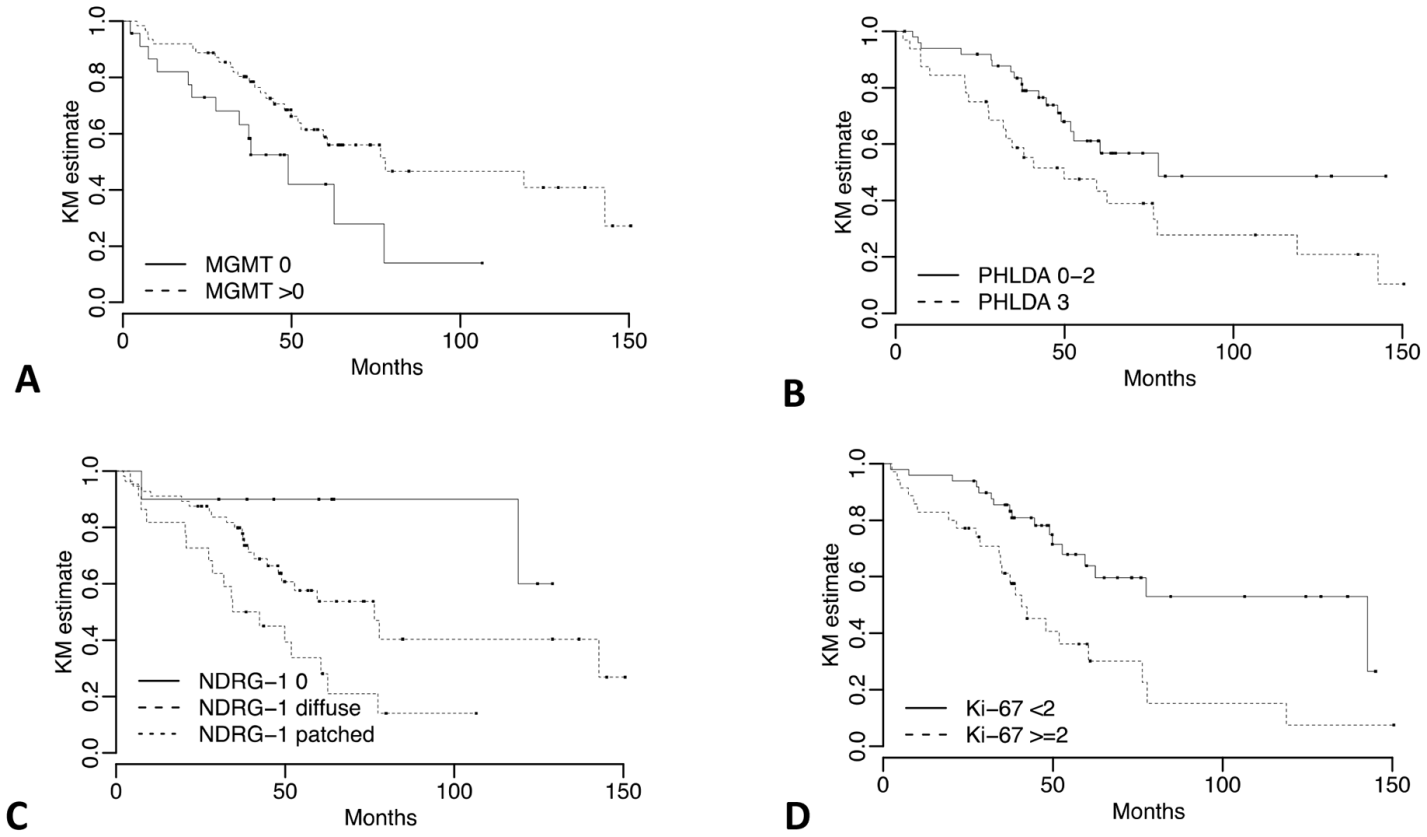
Kaplan-Meier DFS curves for NDRG-1, MGMT, PHLDA-3 and ki-67 IHC expression DFS stratified by **a.** MGMT IHC expression absent vs any positive nuclear staining **b.** PHLDA-3 <50% vs ≥50% nuclear staining **c.** NDRG-1 negative staining vs positive with diffuse pattern vs positive with patched pattern, and **d.** ki-67 <2% vs ≥2%.

Clinical variables including higher ki-67 proliferation index (HR=2.78, 95% CI: 1.48, 5.23; *p = 0.001*) (Figure 1D), larger tumor size (HR=2.36, 95% CI: 1.23,4.54; *p = 0.01*), AJCC classification IIA or higher (HR=3.14, 95% CI: 1.5, 6.58; *p = 0.002*), positive nodal status (HR=2.64, 95% CI: 1.39, 5.02; *p = 0.003*), R1/R2 margin status (HR=3.23, 95% CI: 1.64, 6.36; *p = 7e-04*) and vascular invasion (HR=1.98, 95% CI: 1.04, 3.78; *p = 0.038*) were associated with increased risks in univariate analysis for shorter DFS (Table 3). Only age ≥ 60 years was associated with increased risk of death in the univariate analysis for OS (HR=3.80, 95% CI: 1.26, 11.44, *p = 0.017*).

**Table 3:** Univariate regression models for clinical variables

Variable	DFS					OS				
	N	Median	HR	95% CI	p	N	Median	HR	95% CI	p
**Overall**	92	62.55				92	NR			
**Age** **<60** **≥60**	56 36	62.55 60.52	1.00 0.93	0.5,1.72	0.805	56 36	NR NR	1.00 3.80	1.26,11.4	0.017
**Gender** **Female** **Male**	47 45	77.44 55.85	1.00 1.39	0.77,2.53	0.275	47 45	NR 143.08	1.00 1.52	0.53,4.4	0.437
**Ki-67** **≤2** **>3**	49 35	142.7 40.8	1.002.78	1.48,5.23	0.001	49 35	143.08 NR	1.00 0.72	0.22,2.36	0.591
**AJCC** **1A-1B** **2A-4**	34 57	77.8 47.93	1.00 3.14	1.5,6.58	0.002	34 57	NR 143.08	1.00 2.42	0.67,8.75	0.178
**Size** **<2.5** **≥2.5**	42 49	77.8 47.93	1.00 2.36	1.23,4.54	0.01	42 49	NR 143.08	1.00 1.31	0.43,3.96	0.636
**Nodes Affected** **0** **>0**	45 45	118.8 40.8	1.00 2.64	1.39,5.02	0.003	45 45	NR 143.08	1.00 1.74	0.58,5.2	0.324
**Margin** **R0** **R1/R2**	78 14	77.44 34.56	1.00 3.23	1.64,6.36	7e-04	78 14	NR NR	1.00 1.27	0.35,4.59	0.713
**Vascular Invasion** **No** **Yes**	64 22	76.32 35.02	1.00 1.98	1.04,3.78	0.038	64 22	126.78 NR	1.00 1.58	0.53,4.75	0.414

Descriptive summaries of methylation pattern are given in Table 2. Positive methylation of NDRG-1 in tumor tissue was marginally associated with a decreased risk of DFS (HR=0.55; 95% CI: 0.28, 1.05; *p = 0.071*), while PHLDA-3 methylation in tumor tissue increased the DFS risk (HR=2.21; 95% CI: 0.97, 5.02; *p = 0.058*). NDRG-1 and PHLDA-3 gene methylation was not significantly associated with OS (Table 4).

**Table 4:** Univariate regression models for methylation and different IHC scores

Variable	DFS					OS				
	N	Median	HR	95% CI	p	N	Median	HR	95% CI	p
**Overall**	92	62.55				92	NR			
**MGMT-T** **Methy** **Neg** **Pos**	28 53	NR 60.52	1.00 1.09	0.52,2.29	0.815	28 53	NR NR	1.00 2.06	0.44,9.57	0.356
**NDRG-1-T** **Methy** **Neg** **Pos**	36 41	48.99 77.8	1.00 0.55	0.28,1.05	0.071	36 41	143.08 NR	1.00 1.34	0.45,4.01	0.605
**PHLDA-3-T** **Methy** **Neg** **Pos**	26 55	NR 51.84	1.00 2.21	0.97,5.02	0.058	26 55	NR 143.08	1.00 2.87	0.64,12.8	0.169
**MGMT** **score** **0 (>0%)** **1 (0%)**	62 23	77.8 48.99	1.00 2.31	1.19,4.48	0.013	62 23	NR 126.78	1.00 1.60	0.53,4.82	0.399
**NDRG-1** **score** **0 (0%)** **1 (Diffuse)** **2 (Patched)**	10 56 22	NR 76.32 38.44	1.00 2.85 6.37	0.67,12.1 1.45,27.9	0.005	10 56 22	NR NR 126.78	1.00 0.71 4.05	0.08,6.37 0.5,32.6	0.013
**PHLDA-3** **Score** **0 (<51%)** **1 (≥ 51%)**	50 32	77.8 49.87	1.00 1.94	1.05,3.6	0.036	50 32	NR NR	1.00 0.95	0.032,2.8	0.929

For NDRG-1 IHC, two aspects: intensity of stain and the pattern (diffuse or patched) were combined. Patients with negative NDRG-1, and consequently no discernable pattern, were given the lowest score (0), patients with a positive score and a diffuse pattern were intermediate (1), and patients with a positive score and patched pattern were highest (2). The median DFS was not reached for patients with no staining and no discernable pattern (score 0), versus 76.32 and 38.44 months for patients with positive staining and diffuse pattern (score 1) (HR: 2.85; 95% CI: 0.67, 12.18) and patients with positive staining and patched pattern (score 2) (HR=6.37; 95% CI: 1.45, 27.95), respectively (Figure 1C and Table 4). Similarly, significant differences were observed for DFS if any positive MGMT staining was present (median: 77.8 months) versus those cases with negative staining (median: 48.99 months) (*p*=0.013, Figure 1A and Table 4). Finally, there was a statistically significant difference between ≥ 51% positive nuclei of the tumor cells (median DFS: 49.87 months) for PHLDA-3 versus those with less than 51% (median DFS: 77.8 months) (*p*=0.036, Figure 1B and Table 4).

Only significant differences was observed for OS with the NDRG-1 IHC score (*p = 0.013*, Table 4).

### Immunohistochemistry prognostic score (IPS)

**Figure 2:**
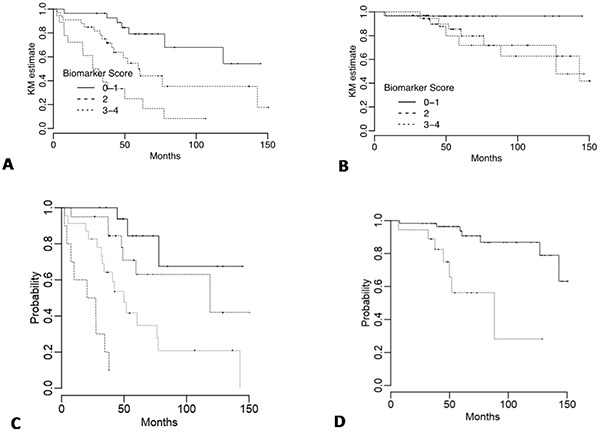
Kaplan-Meier DFS and OS curves for immunohistochemistry (IPS) and overall prognostic factor scores (PFS) DFS and OS for **A.** and **B.** IPS categories with the score calculated as I(MGMT 0%) + I(NDRG-1 di_use) + 2 × I(NDRG-1 patched) + I(PHLDA-3 >50%) where the value for I() is one if the patient's IPS value meets the criterion in parentheses and zero otherwise, and **C-D.** PFS groups derived from the multivariable Cox models (Table 5). Cases are ranked by score and divided into quartiles for DFS and tertiles (first and second vs third) for OS.

An overall IPS was calculated (summarized in Statistical Methods) with individual marker scores coded such that the higher risk categories were greater than zero, and the overall IPS taken as the sum. Overall scores ranged from 0 to 4 with a median of 2. Scores of 0 and 1 were recorded as IPS=0, a score of 2 as IPS=1 and scores 3 and 4 as IPS=2. Kaplan-Meier curves for the IPS are shown in Figure 2A and 2B.

**Table 5:** Multivariate regression model

Variable	DFS				OS			
	Coef	HR	95% CI	p	Coef	HR	95% CI	p
**AJCC** **2A-4**	0.57	1.77	0.76,4.12	0.19	1.01	2.73	0.68,10.92	0.16
**Size** **≥2.5**	0.71	2.04	0.89,4.67	0.09				
**Ki-67** **>3**	0.90	2.45	1.20,5.01	0.01				
**Age** **≥60**					2.04	7.67	2.14,27.45	0.0017
**IPS**	0.99	2.68	1.60,4.49	0.00018	0.98	2.67	1.11,6.41	0.03

**Abbreviations:** Coef: Coefficient

Only significant variables in univariate analysis were included in this Table

### Multivariate analysis

In multivariate analysis ki-67 (HR=2.45; 95% CI: 1.20, 5.01; *p = 0.01*) and the IPS (*p = 0.00018*, HR=2.68; 95% CI: 1.60, 4.49) were independent prognostic factors for DFS (Table 5). Age (*p = 0.0017*, HR=7.67; 95% CI: 2.14, 27.45) and the IPS (*p = 0.03*, HR=2.67; 95% CI: 1.11, 6.41) were independent prognostic factors for OS (Table 5). In both models, the effect of IHC was maintained after adjusting for AJCC classification, and in the DFS model size of the tumor was also adjusted for.

### Discriminatory ability of the Prognostic Factor Score (PFS) models

Our proposed model was tested for recurrence and survival prognostication validity as measured by discrimination by Harrell's c-index (HCI). HCI for the multivariate DFS model with clinical factors only (AJCC classification, margin status, size, and Ki-67) and clinical factors plus IPS were 0.724 and 0.796 respectively. Corrected for optimism by internal validation, these values were 0.703 and 0.780. The modified c-index values for the clinical and clinical plus biomarker models were: 0.704 and 0.786, respectively. The improvement with the addition of the biomarker predictor was 0.082 (95% CI: 0.006, 0.158), which is statistically significant. The formula for calculating an overall PFS for DFS and OS are summarized in Statistical Methods section. Patients were grouped based on quartiles of the PFS and Kaplan-Meier curves plotted in Figure 2C.

HCI for the multivariate OS model with clinical factors only (AJCC classification and age), and clinical factors plus the biomarker score were 0.766 and 0.788 respectively. Corrected for optimism by internal validation, these values were 0.747 and 0.757. The modified c-index values for the clinical and clinical plus biomarker models were: 0.782 and 0.807 respectively. However the improvement with the addition of the biomarker predictor was 0.025 (95% CI: -0.098, 0.148) which is not statistically significant. Patients were divided based on the third quartile of the PFS and Kaplan-Meier curves plotted in Figure 2D.

## DISCUSSION

To the best of our knowledge, this is the first study that combines a panel of genes relevant in PanNET tumorigenesis and clinical-pathological parameters. Our results identify potential prognostic biomarkers for recurrence and survival after surgical resection. We have described the prevalence and prognostic implications of MGMT, NDRG-1 and PHLDA-3 by IHC and methylation in patients with resected PanNET. We then integrated these results and developed a prognostic score to identify patients at higher risk of recurrence and death from PanNET.

We showed that *MGMT IHC* expression has prognostic significance in univariate analysis for DFS in agreement with recently published results in PanNET [19].

PHLDA-3 acts as p53-regulated repressor of AKT [25], controlling the regulatory crosstalk between both proteins and competes with the PH domain of AKT for binding of membrane lipids, therefore, hindering its activation and inducing apoptosis. High frequencies of chromosomal loss and abnormal PHLDA-3 expression have been described in lung neuroendocrine tumors [25]. Our study showed a strong correlation between DFS and PHLDA-3 nuclear expression, with higher expression of PHLDA-3 associated with worse clinical outcomes. However, we identified a reverse correlation between cytoplasmic and nuclear expression of PHLDA-3 in most of the cases (data not showed), which could explain the differences between our findings and previously published results [24]. We hypothesize that loss of heterozygosity could be in correlation with low cytoplasmic PHLDA-3 expression.

NDRG-1 is induced by p53 [28,29] and one of its functions is to control the negative feedback-loop between PTEN and PI3K pathway. We showed using IHC that lower NDRG-1 expression is associated with better clinical outcomes and has a strong influence over DFS and OS. Although, similar findings have been described in other cancers [30,31], this is the first study to show the implications of NDRG-1 expression in patients with PanNET.

We developed a unique IPS score based on IHC MGMT, PHLDA-3 and NDRG-1 expressions, which seems to have a strong prognostic role for DFS and OS in patients with resected PanNET. Not only was the IPS score retained in multivariate DFS and OS analyses, but it was also shown that this factor significantly improved the discrimination of a multivariate DFS containing clinical variables only. This is particularly noteworthy since the insensitivity of the HCI statistic to small changes when assessing overall risk prediction adequacy has been well documented previously [32]. We propose this novel IPS score and our final PFS to identify patients at higher risk of disease recurrence and death following surgical resection. Furthermore, our results could be considered at least as significant as other prognostic models, including the ENETS or the AJCC staging systems [33].

One of the challenges of measuring methylated DNA is its low specificity compared with genomic alterations. In some cases methylation changes can occur in both, the tumor and its surrounding area, as we found in our study (data not showed by Table 2) [34,35]. Although mutations in chromatin-remodeling genes have a crucial role in PanNET [12], it can be considered a neoplastic entity with low number of methylated genes as compared to other neoplasms [36]. A novel comprehensive genome-scale analysis with five PanNET and other neoplastic entities showed that DNA methylation patterns in PanNET cases were very different from all other tumor types analyzed [37]. It could be possible that other genetic alterations are more relevant in PanNET carcinogenesis than methylation patterns, or at least the methylation changes could masquerade different functions than have been described in other neoplasms.

NDRG-1 and PHLDA-3 are both involved in mTOR pathway. NDRG-1 is a downstream effector of mTORC2/SGK1 pathway and a potential target for the mTOR-inhibitor everolimus. PHLDA-3 inhibits the mTOR pathway acting as a repressor of AKT. Therefore, we believed that it would be important to analyze the role of both genes as predictive biomarkers for response to treatment. Patients with higher IHC score for NDRG-1 and PHLDA-3 could have more benefit from treatments with mTOR inhibitors. In addition, patients with intact MGMT expression could profit even more with treatment regimens with mTOR inhibitors because they usually have less benefit with temozolomide-based schemes [38].

There are several limitations of our study that should be recognized. First, even though measures of discrimination were presented and internally validated, it is still necessary to conduct prospective studies to externally validate our findings. Second, our cohort included patients only treated at our institution during 13 years. Although there should be no significant differences in surgical approach among institutions, this warrants validation at external sites as well.

In conclusion, we developed a novel and useful prognostic score that could help to identify patients at higher risk of progression following surgical resection for PanNET. Our findings may be a platform to develop future biomarker driven trials in the adjuvant setting for patients with resected PanNET.

## MATERIALS AND METHODS

### Eligibility criteria

The study was approved by the Institutional Review Board at Johns Hopkins Hospital. Patients who underwent surgical resection for PanNET at Johns Hopkins Hospital from 1998 to 2010 and had documented recurrence or completed follow up >24 months were included in the study. Clinical information and patients’ demographics were obtained from the electronic record system including age, sex, date of surgery, pathology, staging, and overall survival. Grade of tumor based on Ki-67 proliferation index was evaluated according to the ENETS guidelines [10].

### DNA preparation for NDRG-1, MGMT and PHLDA-3 methylation analyses

Genomic DNA was extracted according to standard protocols and quantified using a Nano-Drop spectrophotometer. Up to 3μg of extracted genomic DNA was used for bisulfite treatment. Bisulfite conversion kit (EZ DNA Methylation™ Kit, Zymo Research) was utilized. DNA methylation patterns for CpG island of NDRG-1, MGMT and PHLDA-3 were determined by chemical modification of unmethylated but not the methylated cytosines to uracil. Human T cell lymphocyte (CpG Methylated Jurkat Genomic DNA, New England Biolabs N4002S) and Human Genomic male DNA (Promega, G1471) were used as fully methylated and unmethylated controls, respectively.

For each 25 μl PCR, 2 μl bisulphite-converted DNA was used plus 2.5 μl Buffer (0.332 μl (NH_4_)_2_SO_4_plus 0.67 μl Tris, pH 8.8 plus 0.134 μl MgCl_2_ plus 0.014 μl β-mercaptoethanol and 0.85 μl purified H_2_0), 0.2 μl of Platinum Taq DNA polymerase (10966034 Life Technologies), 0.42 μl of each forward and reverse primer (10μM) for a final concentration of 0.168 μM, 1μl EVA Green, 2.5 μl Fluoroscein (100 nM) for a final concentration of 10 nM and 16.41μl DNase-free water were used. As a control for efficiency of bisulphite conversion, primers for a sequence of β-actin, but representing unconverted genomic DNA, were used (ActG). In contrast to ActB primers, the ActG primer pair recognizes the β-actin sequence from genomic DNA and thus can detect insufficient bisulphite conversion. Amplifications were carried out on an Applied Biosystems Step One Plus system (Applied Biosystems, Foster City, CA). Primer Sequences are specified in Supplementary Information.

Real Time PCRs (qPCR) were done in two replicates of each sample and the C_T_ values of each sample was automatically calculated, with holding conditions of 95°C for 5 min, followed during 40 cycles of 95°C for 30 seconds (melting step), annealing step of 60°C (NDRG-1) or 61°C (MGMT, PHLDA-3 and β-actin) for 30 seconds, extension step of 72°C for 30 s and readout step of 82°C (NDRG-1), 80°C (MGMT, PHLDA-3) and 78°C (β-actin) for 30 seconds with data acquisition after each cycle. Melt curve stage consisted of 95°C for 15 seconds, then down to 60°C for 60 seconds and finally up to 95°C for 15 seconds again. At the end, melting curve analysis checked properties of real-time PCR conditions and amplification products. The percentage of methylation was calculated according to multiply two exponential the difference between C_T_ means for β-actin and the corresponding gene.

### IHC analysis for NDRG1, MGMT and PHLDA-3

**Figure 3:**
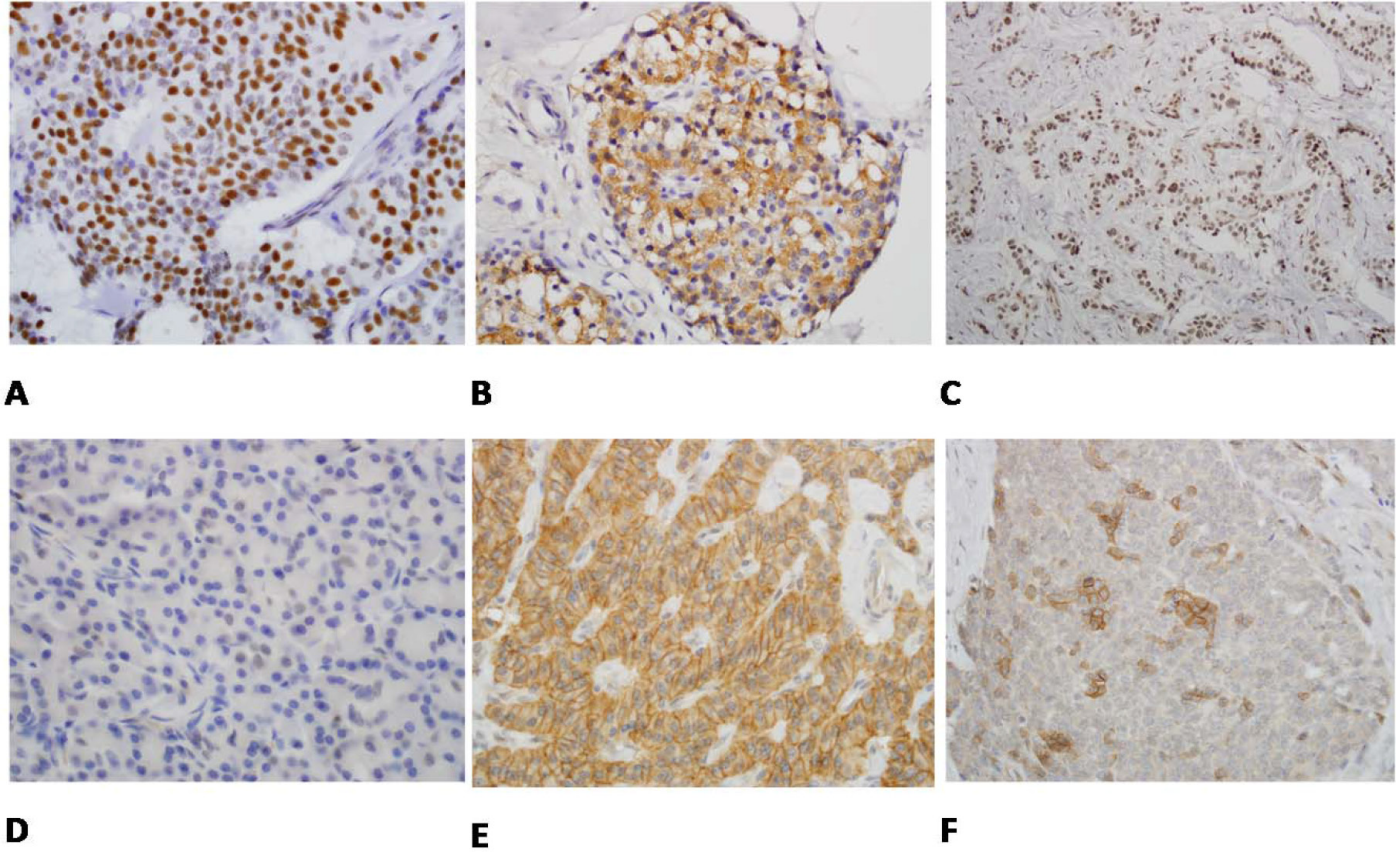
**A.** The tumor cells show strong nuclear staining (3/3) with PHLDA3 immunostain (immunostain × 400). **B.** PHLDA3 immunostain exclusively stains only cytoplasm of tumor cells not the nuclei (3/0) (immunostain × 400). **C.** MGMT immunostain highlights almost all the nuclei of tumor cells (3/3) (immunostain × 400). **D.** MGMT immunostain shows weak and scattered staining of nuclei of the tumor cells (immunostain × 400). **E.** NDRG1 immunostain shows that the tumor cells exhibit diffuse and strong cytoplasmic and membranous staining (3/3) (immunostain × 400). **F.** NDRG1 immunostain shows cytoplasmic and nuclear staining of tumor cells in individual cells and small clusters (2/3) (immunostain × 400).

The H&E slides of all surgically resected specimens were reviewed by doctors ZM and AV. Representative sections of both tumor and benign pancreatic tissue were selected for each case. Three sections (5 μm thick) were cut from PanNET cell blocks of each case, deparaffinized, rehydrated, and subjected to heat-induced antigen retrieval in DAKO Target Retrieval Solution for 40 min. Nonspecific binding sites were blocked with serum-free blocking reagent (DAKO) and NDRG-1 (Goat anti-NDRG1, Abcam, Cambridge, England, UK 1:1000), MGMT (Mouse anti-MGMT monoclonal antibody, Abcam, Cambridge, England, UK 1:100) and PHLDA-3 (Rabbit anti-PHLDA3, Sigma-Aldrich, Saint Louis, USA 1:200) were applied overnight at 4°C on three separate slides for each case. Immunodetection was carried out with horseradish peroxidase (HRP)-conjugated anti-goat, anti-mouse or anti-rabbit (DAKO) for 40 min and 3,3′-Diaminobenzidine was then used as the visualizing substrate. Finally, sections were counterstained with hematoxylin. The percentage of NDRG-1-, MGMT-, and PHLDA3-positive tumor cells per lesion was determined by semiquantitative assessment of the entire tumor section. NDRG-1 and PHLDA-3 cytoplasmic staining intensity was scored from 0 (no staining) to + 3 (strong staining). The staining pattern (diffuse *versus* patched) was also evaluated. Using the percentage of tumor cells showing nuclear immunoreactivity to MGMT and PHLDA-3, we established a scoring system with 0 (no staining in tumor cells), +1 (≤5% positive tumor cells), +2 (6–50% positive tumor cells), and +3 (≥ 51% positive tumor cells) (Figure 3). To rule out false-positive staining, we initially tested several antibodies dilutions in cell-block sections of LnCap and H1299, which are cell lines with known high and low expressions of NDRG-1, MGMT and PHLDA-3. Ki-67 immunostaining were performed for those cases that did not have Ki-67 in their profile. Ki-67 IHC were reviewed for all cases and scored as follows: ≤2%, 3%- 19%, and ≥20%.

### Statistical analysis

The primary statistical objective of the study was to evaluate methylation and IHC expression of MGMT, PHLDA-3 and NDRG-1 as prognostic biomarkers for disease free survival (DFS) and overall survival (OS). DFS was calculated from the time of surgery to date of relapse, death, or last follow-up and OS was calculated from date of surgery to date of death or last follow-up. Event time distributions for DFS and OS were estimated with the method of Kaplan and Meier and, compared using the log-rank statistic, or the Cox proportional-hazards regression model. For methylation variables a cut-off was established based on the percentage of methylation for each gene < median (non-methylated) and ≥ median (methylated) according to previously published standards [35]. IHC categories for MGMT and PHLDA-3 were collapsed and entered into regression models as binary scores (0 vs non-0 and <51% vs ≥ 51% positive tumor cells for MGMT and PHLDA-3, respectively).

The effect of IPS, adjusting for important clinical factors was evaluated with multivariate Cox proportional-hazards models. All factors that were potentially prognostic when considered alone (*p* value less than 0.15) were entered into the multivariate model. Factors that were not statistically significant were removed with backward elimination and re-estimation of hazard ratios at each step. In some cases known prognostic factors that were marginally significant were retained in the model to demonstrate their effect in the presence of other factors. Prognostic factor score (PFS) models for DFS and OS combining clinical and IPS were based on the final multivariate Cox models.

Discriminatory ability of the PFS models was assessed with two concordance measures: Harrell's bias corrected c-index [39], and a modification of Harrell's c-index proposed by Uno [40]. These concordance measures involve the assessment of all possible pairs of patients to determine the probability that patients with higher predicted risk scores have earlier event times. For studies with long follow-up, HCI estimator may be dependent on the censoring mechanism and therefore the modified concordance index was also calculated. Improvement in discrimination with the inclusion of the IPS to a model with only clinical predictors was assessed with the difference in the modified c-index.

Statistical analysis was performed using the Statistical Analysis System, version 9.1, and R 3.0, and all *p* values reported are two sided.

### Formula to calculate IPS

When calculating an IPS based on IHC, the two aspects of NDRG-1, intensity of stain and the pattern, were combined. Patients with a negative NDRG-1 IHC score (NDRG1 score 0 or 1), and consequently no discernible pattern, had the lowest risk for these endpoints, while patients with a positive score (NDRG1 score 2 or 3) and a diffuse pattern was intermediate, and those with positive scores and a patched pattern were at the highest risk. Individual marker scores were coded such that the higher risk category (categories for NDRG-1) were greater than zero, and the overall score taken as the sum: I (MGMT 0%) + I (NDRG-1 diffuse) + 2xI (NDRG-1 patched) + I (PHLDA-3 ≥ 51). The value for I () is one if the patient's IHC value meets the criterion in parentheses and zero otherwise. Patients with no NDRG-1 IHC stain were coded zero for both pattern types and therefore the NDRG-1 contribution to the biomarker score for these patients was zero. NDRG-1 positive with diffuse pattern increased the overall score by one, while NDRG-1 positive with patched pattern increased the overall score by two.

### Formula to calculate PFS

The formula for calculation an overall PFS for DFS was calculated based on the model in follow (Table 5) as: 0.987 × (IPS) + 0.569 × I (AJCC classification 2-4) + 0.712 × I (size ≥2.5) + 0.897 × I (Ki-67 >3%).

An overall PFS for OS was calculated based on the model in follow (Table 5) as: 0.982 × (IPS) + 2.037 × I (age ≥60) + 1.005 × I (AJCC classification 2-4).

## SUPPLEMENTARY INFORMATION TABLE



## References

[1] Metz DC, Jensen RT (2008). Gastrointestinal neuroendocrine tumors: pancreatic endocrine tumors. Gastroenterology.

[2] Taal BG, Visser O (2004). Epidemiology of neuroendocrine tumours. Neuroendocrinology.

[3] Eriksson B, Oberg K (2000). Neuroendocrine tumours of the pancreas. Br J Surg.

[4] Falconi M, Plockinger U, Kwekkeboom DJ, Manfredi R, Korner M, Kvols L, Pape UF, Ricke J, Goretzki PE, Wildi S, Steinmuller T, Oberg K, Scoazec JY (2006). Well-differentiated pancreatic nonfunctioning tumors/carcinoma. Neuroendocrinology.

[5] Yao JC, Hassan M, Phan A, Dagohoy C, Leary C, Mares JE, Abdalla EK, Fleming JB, Vauthey JN, Rashid A, Evans DB (2008). One hundred yerars after “carcinoid”: epidemiology of and prognostic factors for neuroendocrine tumors in 35,825 cases in United States. J Clin Oncol.

[6] Hill JS, McPhee JT, McDade TP, Zhou Z, Sullivan ME, Whalen GF, Tseng JF (2009). Pancreatic neuroendocrine tumors: the impact of surgical resection on survival. Cancer.

[7] Schurr PG, Strate T, Rese K, Kaifi JT, Reichelt U, Petri S, Kleinhans H, Yekebas EF, Izbicki JR (2007). Aggressive surgery improves long-term survival in neuroendocrine pancreatic tumors: an institutional experience. Ann Surgery.

[8] Oberg K (2012). Neuroendocrine tumors of the digestive tract: impact of new classifications and new agents on therapeutic approaches. Curr Opin Oncol.

[9] Bosman F, Carneiro F, Hubran R (2010). WHO classification of tumours of the digestive system.

[10] Rindi G, Klöppel G, Couvelard A, Komminoth P, Körner M, Lopes JM, McNicol AM, Nilsson O, Perren A, Scarpa A, Scoazec JY, Wiedenmann B (2007). TNM staging of midgut and hindgut (neuro) endocrine tumors: A consensus proposal including a grading system. Virchows Arch.

[11] Young K, Iyer R, Morganstein D, Chau I, Cunningham D, Starling N (2015). Pancreatic neuroendocrine tumors: a review. Future Oncol.

[12] Jiao Y, Shi C, Edil BH, de Wilde RF, Klimstra DS, Maitra A, Schulick RD, Tang LH, Wolfgang CL, Choti MA, Velculescu VE, Diaz LA, Vogelstein B (2011). DAXX/ATRX, MEN1, and mTOR pathway genes are frequently altered in pancreatic neuroendocrine tumors. Science.

[13] Strosberg JR, Fine RL, Choi J, Nasir A, Coppola D, Chen DT, Helm J, Kvols L (2011). First-line chemotherapy with capecitabine and temozolomide in patients with metastatic pancreatic endocrine carcinomas. Cancer.

[14] Yao JC, Shah MH, Ito T, Bohas CL, Wolin EM, Van Cutsem E, Hobday TJ, Okusaka T, Capdevila J, de Vries EG, Tomassetti P, Pavel ME, Hoosen S (2011). Everolimus for advanced pancreatic neuroendocrine tumors. N Engl J Med.

[15] Valle JW, Eatock M, Clueit, Gabriel Z, Ferdinand R, Mitchell S (2014). A systematic review of non-surgical treatments for pancreatic neuroendocrine tumours. Cancer Treat Rev.

[16] Jacinto FV, Esteller M (2007). MGMT hypermethylation: a prognostic foe, a predictive friend. DNA Repair (Amst).

[17] Kulke MH, Hornick JL, Frauenhoffer C, Hooshmand S, Ryan DP, Enzinger PC, Meyerhardt JA, Clark JW, Stuart K, Fuchs CS, Redston MS (2009). O6-methylguanine DNA methyltransferase deficiency and response to temozolomide-based therapy in patients with neuroendocrine tumors. Clin Cancer.

[18] Walter T, van Brakel B, Vercherat C, Hervieu V, Forestier J, Chayvialle JA, Molin Y, Lombard-Bohas C, Joly MO, Scoazec JY (2015). O(6)-Methylguanine-DNA methyltransferase status in neuroendocrine tumours: prognosis relevance and association with response to alkylating agents. Br J Cancer.

[19] Schmitt AM, Pavel M, Rudolph T, Dawson H, Blank A, Komminoth P, Vassella E, Perren A (2014). Prognosis and predictive role of MGMT protein expression and promoter methylation in sporadic pancreatic neoplasm. Neuroendocrinoloy.

[20] Kovacevic Z, Chikhani S, Lui GY, Sivagurunathan S, Richardson DR (2013). The iron-regulated metastasis suppressor NDRG1 targets NEDD4L, PTEN, and SMAD4 and inhibits the PI3K and Rassignaling pathways. Antioxid Redox Signal.

[21] Missiaglia E, Dalai I, Barbi S, Beghelli S, Falconi M, della Peruta M, Piemonti L, Capurso G, Di Florio A, delle Fave G, Pederzoli P, Croce CM, Scarpa A (2010). Pancreatic endocrine tumors: expression profiling evidences a role for AKT-mTOR pathway. J Clin Oncol.

[22] Lachat P, Shaw P, Gebhard S, van Belzen N, Chaubert P, Bosman FT (2008). Expression of NDRG1, a differentiation-related gene, in human tissues. Histochem Cell Bio.

[23] Blaes J, Weiler M, Sahm F, Osswald M, Czabanka M, Thomé CM, Schliesser MG, Pusch S, Luger S, Winkler F, Radbruch A, Jugold M, Simon M (2014). NDRG1 prognosticates the natural course of disease in WHO grade II glioma. J Neurooncol.

[24] Ohki R, Saito K, Chen Y, Kawase T, Hiraoka N, Saigawa R, Minegishi M, Aita Y, Yanai G, Shimizu H, Yachida S, Sakata N, Doi R (2014). PHLDA3 is a novel tumor suppressor of pancreatic neuroendocrine tumors. Proc Natl Acad Sci USA.

[25] Kawase T, Ohki R, Shibata T, Tsutsumi S, Kamimura N, Inazawa J, Ohta T, Ichikawa H, Aburatani H, Tashiro F, Taya Y (2009). PH domain-only protein PHLDA3 is a p53-regulated repressor of Akt. Cell.

[26] Muroi H, Nakajima M, Satomura H, Takahashi M, Yamaguchi S, Sasaki K, Yokobori T, Miyazaki T, Kuwano H, Kato H (2015). Low PHLDA3 expression in oesophageal squamous cell carcinoma is associated with poor prognosis. Anticancer Res.

[27] Yoo NJ, Kim YR, Lee SH (2011). Expressional and mutational analysis of PHLDA3 gene in common human cancers. Pathology.

[28] Stein S, Thomas EK, Herzog B, Westfall MD, Rocheleau JV, Jackson RS, Wang M, Liang P (2004). NDRG1 is necessary for p53-dependent apoptosis. J Biol Chem.

[29] Zhang AH, Rao JN, Zou T, Liu L, Marasa BS, Xiao L, Chen J, Turner DJ, Wang JY (2007). p53-dependent NDRG1 expression induces inhibition of intestinal epithelial cell proliferation but not apoptosis after polyamine depletion. Am J Physiol Cell Physiol.

[30] Nishio S, Ushijima K, Tsuda N, Takemoto S, Kawano K, Yamaguchi T, Nishida N, Kakuma T, Tsuda H, Kasamatsu T, Sasajima Y, Kage M, Kuwano M (2008). Cap43/NDRG1/Drg-1 is a molecular target for angiogenesis and a prognostic indicator in cervical adenocarcinoma. Cancer Lett.

[31] Akiba J, Ogasawara S, Kawahara A, Nishida N, Sanada S, Moriya F, Kuwano M, Nakashima O, Yano H (2008). N-myc downstream regulated gene 1 (NDRG1)/Cap43 enhances portal vein invasion and intrahepatic metastasis in human hepatocellular carcinoma. Oncol Rep.

[32] Pepe MS, Cai T (2004). The analysis of placement values for evaluating discriminatory measures. Biometrics.

[33] Ellison TA, Wolfgang CL, Shi C, Cameron JL, Murakami P, Mun LJ, Singhi AD, Cornish TC, Olino K, Meriden Z, Choti M, Diaz LA, Pawlik TM (2014). A single institution's 26-year experience with nonfunctional pancreatic neuroendocrine tumors: a validation of current staging systems and a new prognostic nomogram. Ann Surg.

[34] Karayan-Tapon L, Quillien V, Guilhot J, Wager M, Fromont G, Saikali S, Etcheverry A, Hamlat A, Loussouarn D, Campion L, Campone M, Vallette FM, Gratas-Rabbia-Ré C (2010). Prognostic value of O6-methylguanine-DNA methyltransferase status in glioblastoma patients, assessed by 5 different methods. J Neurooncol.

[35] Li M, Chen WD, Papadopoulos N, Goodman SN, Bjerregaard NC, Laurberg S, Levin B, Juhl H, Arber N, Moinova H, Durkee K, Schmidt K, He Y (2009). Sensitive digital quantification of DNA methylation in clinical samples. Nat Biotechnol.

[36] Stefanoli M, La Rosa S, Sahnane N, Romualdi C, Pastorino R, Marando A, Capella C, Sessa F, Furlan D (2014). Prognostic Relevance of Aberrant DNA Methylation in G1 and G2 Pancreatic Neuroendocrine Tumors. Neuroendocrinology.

[37] Timp W, Bravo HC, McDonald OG, Goggins M, Umbricht C, Zeiger M, Feinberg AP, Irizarry RA (2014). Large hypomethylated blocks as a universal defining epigenetic alteration in human solid tumors. Genome Medicine.

[38] Weiler M, Blaes J, Pusch S, Sahm F, Czabanka M, Luger S, Bunse L, Solecki G, Eichwald V, Jugold M, Hodecker S, Osswald M, Meisner C (2014). mTOR target NDRG1 confers MGMT-dependent resistance to alkylating chemotherapy. Proc Natl Acad Sci U S A.

[39] Harrell FE, Califf RM, Pryor DB, Lee KL, Rosati RA (1982). Evaluating the yield of medical tests. JAMA.

[40] Uno H, Cai T, Pencina MJ, D'Agostino RB, Wei LJ (2011). On the C-statistics for evaluating overall adequacy of risk prediction procedures with censored survival data. Stat Med.

