# Correction: A new immunohistochemistry prognostic score (IPS) for recurrence and survival in resected pancreatic neuroendocrine tumors (PanNET)

**DOI:** 10.18632/oncotarget.16155

**Published:** 2017-03-13

**Authors:** Antonio Viúdez, Filipe L.F. Carvalho, Zahra Maleki, Marianna Zahurak, Daniel Laheru, Alejandro Stark, Nilofer S. Azad, Christopher L. Wolfgang, Stephen Baylin, James G. Herman, Ana De Jesus-Acosta

**Present**: Due to a proofreading error, one of the author’s names was misspelled as: Nilofer Z. Azad.

**Correct**: The proper spelling is as follows: Nilofer S. Azad. The publishers sincerely apologize for this oversight.

Original article: Oncotarget. 2016; 7(18):25950-61. doi: 10.18632/oncotarget.7436.

