# Supplementary material for: A new immunohistochemistry prognostic score (IPS) for recurrence and survival in resected pancreatic neuroendocrine tumors (PanNET)
Source: Oncotarget. 2016 Feb 17;7(18):24950–61. doi: 10.18632/oncotarget.7436 (PMC5041882; doi:10.18632/oncotarget.7436)
Supplement: Supplementary file 1 [file oncotarget-07-24950-s001.pdf]

SUPPLEMENTARY INFORMATION

Primer sequences

MGMT Methylated reactions: 5'-TTT CGA CGT TCG TAG GTT TTC GC-3' (forward) and 5'-GCA CTC TTC CGA AAA CGA AAC G-3' (reverse).

NDRG-1 Methylated reactions: 5'-GAGTCGG AGTAAGTGTTTGCGAGCG-3' (forward) and 5'-CCA AATATCCGCGAAACCCCTCTCG-3' (reverse).

PHLDA-3 Methylated reactions: 5'-GAGGGTC GGTTAGGGTAGGAATGCG-3' (forward) and 5'-TCTT ACTCCCCTAAACTCTATCTACGCG-3' (reverse).

SUPPLEMENTARY TABLE

Supplementary Table S1: Comparison analysis between patients with molecular analyses (YES) and patients in which molecular analyses were not done (NO)

Variable	N	No<br>N=74<br>% (n)	Yes<br>N=92<br>% (n)	Test Statistic
**Age**	166	$_{44}53_{64}$ (54±15)	$_{50}56_{64}$ (57±12)	$F_{1,164}$=2, p=0.1[1]
**Gender**	166			X_1^2=0.7, p=0.4[2]
**Female**		45 (33)	51 (47)	
**Male**		55 (41)	49 (45)	
**Grade**	157			X_2^2=1, p=0.5[2]
**I**		79 (57)	80 (68)	
**II**		18 (13)	14 (12)	
**III**		3 (2)	6 (5)	
**Type**	166			X_5^2=3, p=0.6[2]
**Insulinoma**		4 (3)	4 (4)	
**Glucagonoma**		3 (2)	2 (2)	
**VIPoma**		0 (0)	2 (2)	
**Gastrinoma**		3 (2)	3 (3)	
**Somatostatinoma**		0 (0)	2 (2)	
**Non-functional**		91 (67)	86 (79)	
**Ki-67**	108			X_2^2=1, p=0.5[2]
**<2**		46 (11)	58 (49)	
**2-20**		46 (11)	37 (31)	
**>20**		8 (2)	5 (4)	
**AJCC**	156			X_5^2=8, p=0.2[2]
**1A**		35 (23)	22 (20)	
**1B**		15 (10)	15 (14)	
**2A**		8 (5)	8 (7)	
**2B**		29 (19)	36 (33)	
**3**		5 (3)	1 (1)	
**4**		8 (5)	18 (16)	
**Size (cm)**	164	$_{2}2_{5}$ (4±3)	$_{2}2_{4}$ (4±3)	$F_{1,162}$=0.07, p=0.8[1]

(*Continued*)

Variable	N	No<br>N=74<br>% (n)	Yes<br>N=92<br>% (n)	Test Statistic
**Nodes Affected**	166			X_2^2=1, p=0.3[2]
**Yes**		59 (43)	50 (45)	
**No**		41 (30)	50 (45)	
**Margin**	166			X_2^2=0.2, p=0.9[2]
**R0**		85 (63)	85 (78)	
**R1**		14 (10)	13 (12)	
**R2**		1 (1)	2 (2)	
**Vascular Invasion**	153			X_1^2=1, p=0.2[2]
**Yes**		34 (23)	26 (22)	
**No**		66 (44)	74 (64)	
**Perineural Invasion**	155			X_1^2=2, p=0.2[2]
**Yes**		28 (19)	39 (34)	
**No**		72 (48)	61 (54)	
**Period Recluiment**	166			X_1^2=6, p=0.01[3]
**1998-2001**		12 (9)	17 (16)	
**2001-2005**		5 (4)	23 (21)	
**2005-2009**		34 (25)	25 (23)	
**2009-2012**		49 (36)	35 (32)	

Because of the requirement for at least two years of follow-up, patients not included in this study were more likely to have had surgery in the later time periods, between 2005 and 2010. The median follow-up in this group was substantially shorter (45 months versus 60 months) (data not showed). Median DFS in the study group was 63 months, and the five-year OS was 84.5% (95% CI: 76-94%).
